# Effects of the group I metabotropic glutamate receptor agonist, DHPG, and injection stress on striatal cell signaling in food-restricted and ad libitum fed rats

**DOI:** 10.1186/1471-2202-5-50

**Published:** 2004-12-03

**Authors:** Yan Pan, Yemiliya Berman, Kenneth D Carr

**Affiliations:** 1 Department of Psychiatry, Millhauser Laboratories, New York University School of Medicine, 550 First Ave, New York, NY 10016, USA; 2 Department of Pharmacology, New York University School of Medicine, 550 First Ave, New York, NY 10016, USA

## Abstract

**Background:**

Chronic food restriction augments the rewarding effect of centrally administered psychostimulant drugs and this effect may involve a previously documented upregulation of D-1 dopamine receptor-mediated MAP kinase signaling in nucleus accumbens (NAc) and caudate-putamen (CPu). Psychostimulants are known to induce striatal glutamate release, and group I metabotropic glutamate receptors (mGluR) have been implicated in the cellular and behavioral responses to amphetamine. The purpose of the present study was to evaluate whether chronic food restriction increases striatal MAP kinase signaling in response to the group I mGluR agonist, DHPG.

**Results:**

Western immunoblotting was used to demonstrate that intracerebroventricular (i.c.v.) injection of DHPG (500 nmol) produces greater activation of ERK1/2 and CREB in CPu and NAc of food-restricted as compared to ad libitum fed rats. Fos-immunostaining induced by DHPG was also stronger in CPu and NAc core of food-restricted relative to ad libitum fed rats. However, i.c.v. injection of saline-vehicle produced greater activation of ERK1/2 and CREB in CPu and NAc of food-restricted relative to ad libitum fed rats, and this difference was not seen when subjects received no i.c.v. injection prior to sacrifice. In addition, although DHPG activated Akt, there was no difference in Akt activation between feeding groups. To probe whether the augmented ERK1/2 and CREB activation in vehicle-injected food-restricted rats are mediated by one or more GluR types, effects of an NMDA antagonist (MK-801, 100 nmol), AMPA antagonist (DNQX, 10 nmol), and group I mGluR antagonist (AIDA, 100 nmol) were compared to saline-vehicle. Antagonist injections did not diminish activation of ERK1/2 or CREB.

**Conclusions:**

These results indicate that a group I mGluR agonist induces phosphorylation of Akt, ERK1/2 and CREB in both CPu and NAc. However, group I mGluR-mediated signaling may not be upregulated in food-restricted rats. Rather, a physiological response to "i.c.v. injection stress" is augmented by food restriction and appears to summate with effects of the group I mGluR agonist in activating ERK1/2 and CREB. While the augmented cellular response of food-restricted rats to i.c.v. injection treatment represents additional evidence of enhanced CNS responsiveness in these subjects, the functional significance and underlying mechanism(s) of this effect remain to be elucidated.

## Background

Chronic food restriction increases central sensitivity to rewarding and motor-activating effects of psychostimulants and direct dopamine receptor agonists [1]. Corresponding adaptive changes at the cellular level include increased psychostimulant-induced DA release in nucleus accumbens core [2], and upregulation of D-1 DA receptor-mediated MAP kinase signaling in dorsal and ventral striatum, with consequent increased activation of CREB and the immediate early gene, *c-fos *[3,4]. Changes in striatal glutamate receptor function have not been investigated but are of interest in light of findings that (i) psychostimulants induce striatal glutamate release [5-7], (ii) amphetamine-induced activation of striatal MAP kinase, CREB, and *c-fos *is attenuated by a group I metabotropic glutamate receptor antagonist [8], and (iii) the augmented effects of striatal D-1 receptor stimulation in food-restricted rats include hyperphosphorylation of the NMDA receptor NR1 subunit [9].

The group I mGluRs (mGluR1/5) are of particular interest. Group I mGluRs are densely expressed in striatal medium spiny neurons [10] and activate phospholipase C, resulting in hydrolysis of phosphoinositides and activation of Ca^2+^-dependent signaling cascades [11]. The group I mGluR agonist, DHPG, increases the phosphorylation of extracellular signal-regulated kinase (ERK) and the transcription factor CREB when infused into dorsal striatum [12], as do cocaine injected systemically and the D-1 agonist SKF-82958 injected into the lateral ventricle [4,13]. DHPG also elicits hyperlocomotion resembling that induced by DA receptor agonists, and the effect is not attenuated by the D-1 DA receptor antagonist, SCH23390 [10]. It is therefore possible that glutamate and group I mGluR function contribute to the changes in psychostimulant-induced behavioral responses and striatal cell signaling in food-restricted subjects. The purpose of the first experiment of this study was to compare dorsal and ventral striatal cell signaling in response to intracerebroventricular (i.c.v.) administration of the group I mGluR agonist, DHPG, in ad libitum fed and food-restricted rats. A dose of 500 nmol was used based on pilot work and published reports indicating that this dose is sufficient to exert behavioral effects [14] and stimulate PI hydrolysis [11] while being below threshold for producing convulsive behavior [15]. A second experiment was based on results of the first experiment and sought to elucidate the observed increase in striatal ERK1/2 and CREB phosphorylation in food-restricted rats injected i.c.v. with saline-vehicle.

## Results

### Experiment 1

**Figure 1:**
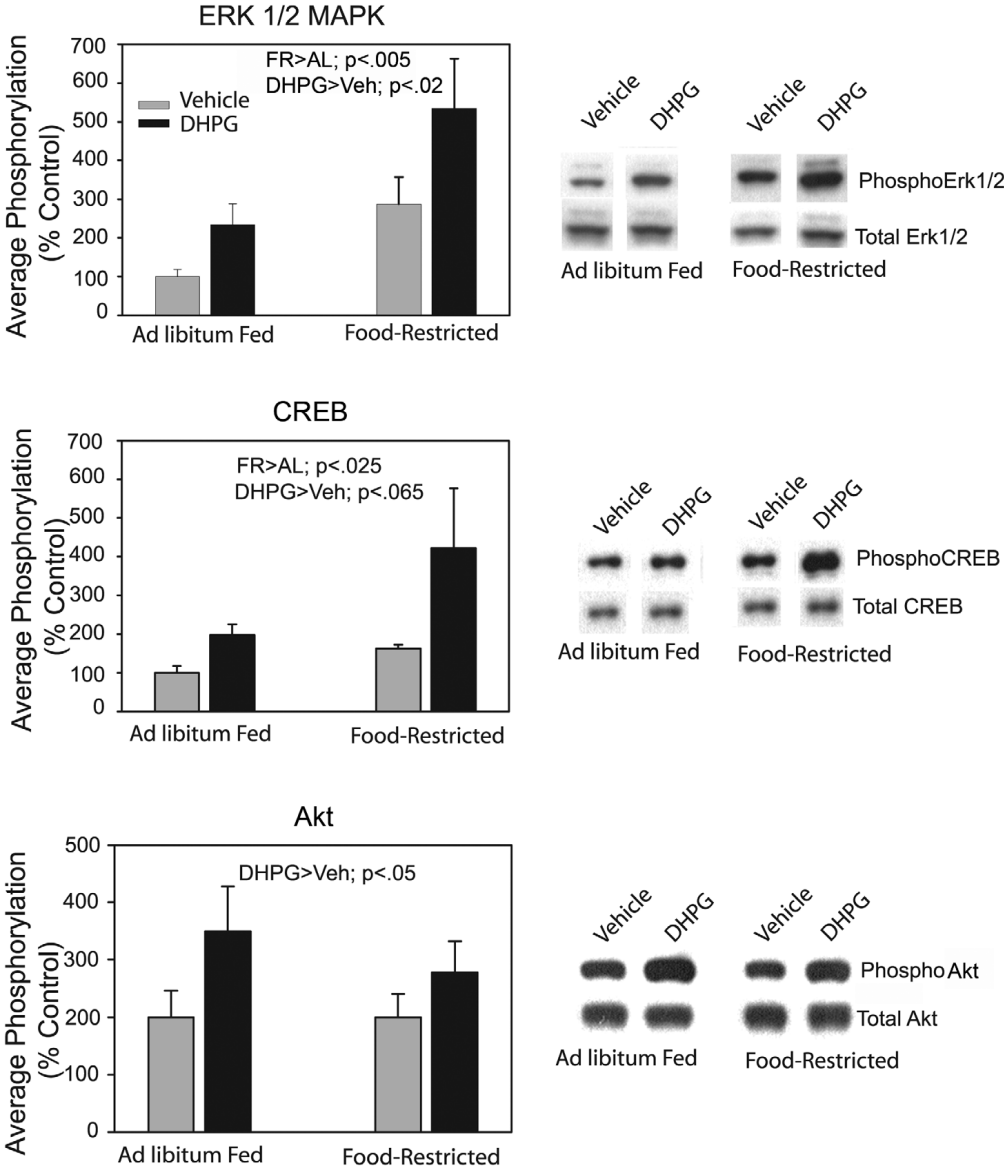
**Effects of DHPG on activation of ERK1/2, CREB and Akt in Caudate-Putamen **Ad libitum fed and food-restricted rats received i.c.v. injections of DHPG (500 nmol) or saline vehicle prior to sacrifice. Lysates were immunoblotted with anti-phospho ERK1/2 or anti-ERK1/2 (top), anti-phospho-CREB or anti-CREB (center), or anti-phospho-Akt or anti-Akt (bottom) antibodies. Following densitometry, intensities of bands corresponding to phosphorylated proteins were divided by intensities of the corresponding total protein bands to correct for small differences in protein loading. Results (mean ± S.E.M.) are expressed in comparison to the normalized control, which was defined as the ad libitum fed group injected with vehicle. Graphed results are displayed with representative immunoblots.

**Figure 2:**
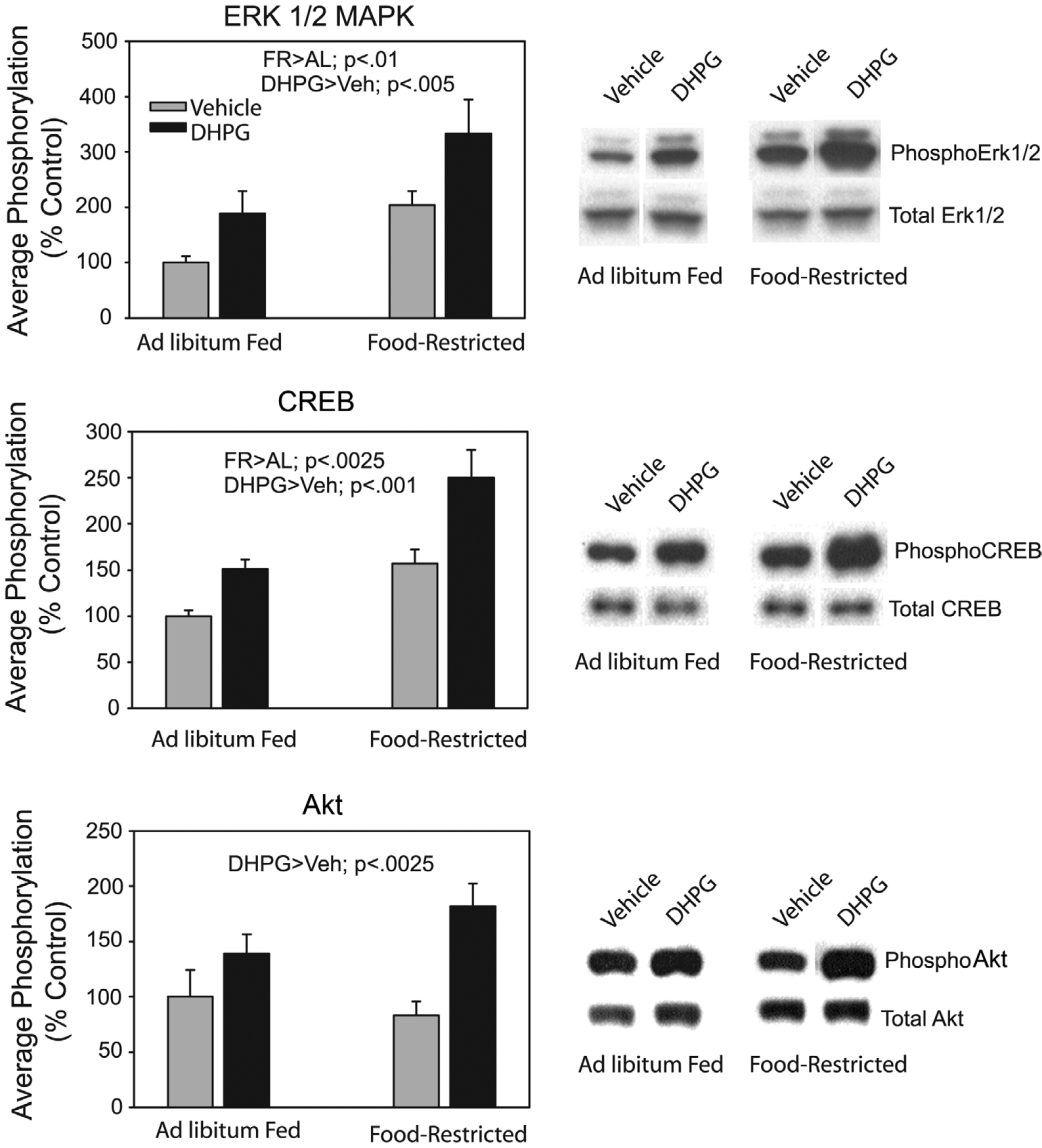
**Effects of DHPG on activation of ERK1/2, CREB and Akt in Nucleus Accumbens **Ad libitum fed and food-restricted rats received i.c.v. injections of DHPG (500 nmol) or saline vehicle prior to sacrifice. Lysates were immunoblotted with anti-phospho ERK1/2 or anti-ERK1/2 (top), anti-phospho-CREB or anti-CREB (center), or anti-phospho-Akt or anti-Akt (bottom) antibodies. Following densitometry, intensities of bands corresponding to phosphorylated proteins were divided by intensities of the corresponding total protein bands to correct for small differences in protein loading. Results (mean ± S.E.M.) are expressed in comparison to the normalized control, which was defined as the ad libitum fed group injected with vehicle. Graphed results are displayed with representative immunoblots.

In both the caudate-putamen and nucleus accumbens ERK1/2 phosphorylation was increased by food restriction (Cpu: F_1,18 _= 10.8, p < .005; NAc: F_1,18 _= 8.2, p < .01) and by DHPG injection (Cpu: F_1,18 _= 6.6, p < .02; NAc: F_1,18 _= 10.6, p < .005). However, there was no interaction between feeding condition and drug treatment in either brain region (Cpu: F_1,18 _= 0.6; NAc: F_1,18 _= 0.3). This analysis supports the impression (see Figures 1 &2, top panel) that food restriction did not actually increase the group I mGluR-mediated response to DHPG; rather, increased signaling in the food-restricted group, irrespective of i.c.v. injection treatment, appears to have summated with DHPG to produce a greater net effect in the food-restricted than ad libitum fed group. An identical pattern of results was obtained for CREB phosphorylation (Figures 1 &2 center panel; Cpu: F_diet; 1,18 _= 6.2, p < .025; F_drug; 1,18 _= 3.9, p = .06; F_diet × drug; 1,18 _= 1.2; NAc: F_diet; 1,18 _= 12.9, p < .0025; F_drug; 1,18 _= 15.3, p < .001; F_diet × drug; 1,18 _= 2.7, p > .10).

Interestingly, Akt phosphorylation was induced by DHPG but did not differ between food-restricted and ad libitum fed groups in either the Cpu (F_drug; 1,18 _= 4.5, p < .05; F_diet; 1,18 _= 0.4; F_diet × drug; 1,18 _= 0.4) or NAc (F_drug; 1,18 _= 12.7, p < .0025; F_diet; 1,18 _= 0.4; F_diet × drug; 1,18 _= 2.4, p > .10; Figures 1 &2 bottom panel).

**Figure 3:**
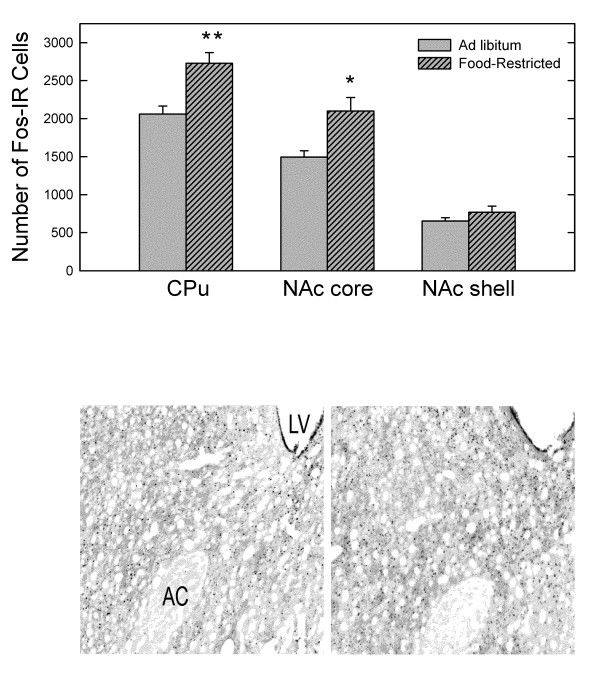
**Effects of DHPG on Fos-like immunostaining in Caudate-Putamen and Nucleus Accumbens core and shell **Mean (±S.E.M.) bilateral number of c-Fos immunoreactive (IR) cells within caudate-putamen (CPu) at coronal levels +1.7 and -0.3 mm (combined) and nucleus accumbens (Nac) core and shell at coronal level +1.5 mm in relation to bregma. Ad libitum fed and food-restricted rats were injected i.c.v. with DHPG (500 nmol) 90 min prior to perfusion. *p < .025, **p < .01 compared to ad libitum fed rats. Representative digital photomicrographs depicting Fos-like immunoreactivity in NAc of ad libitum fed (left) and food-restricted (right) rats are included. LV = lateral ventricle; AC = anterior commissure.

In previous studies, striatal Fos-immunostaining was greater in food-restricted relative to ad libitum fed rats injected i.c.v. with d-amphetamine or the D-1 DA receptor agonist SKF-82958, but not saline-vehicle [3,16]. Therefore, in a small sample of ad libitum fed and food-restricted subjects, brains were processed for Fos-immunostaining and revealed stronger DHPG-induced staining in CPu (t(7) = 3.6, p < .01) and NAc core (t(7) = 2.8 p < .025) but not NAc shell (t(7) = 1.1) of food-restricted, relative to ad libitum fed, subjects (see Figure 3).

During the period between DHPG injection and sacrifice, rats, which remained in their home cages, did not display any obvious behavioral responses to the drug.

### Experiment 2

**Figure 4:**
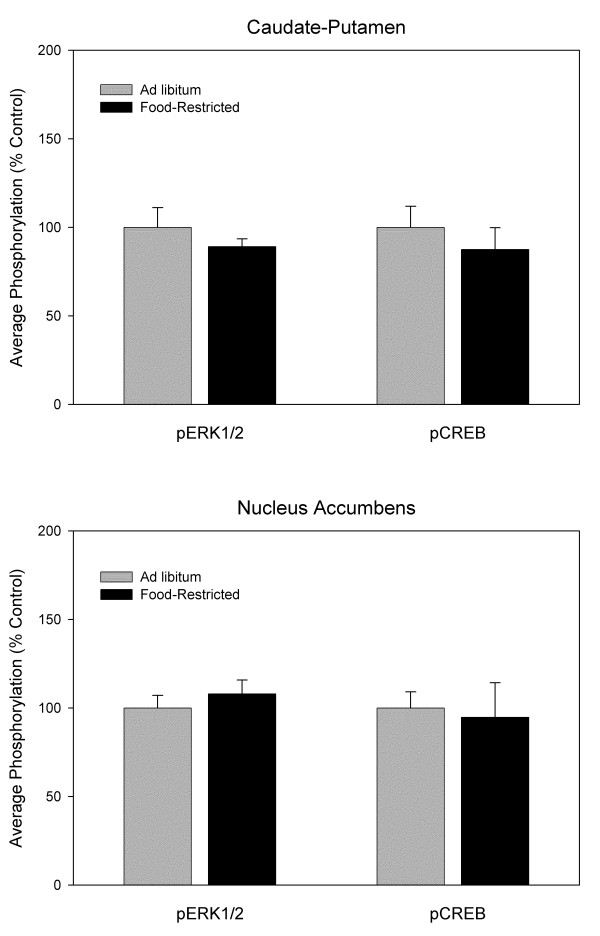
**Basal levels of phospho-ERK1/2 and -CREB in Caudate-Putamen and Nucleus Accumbens **Ad libitum fed and food-restricted rats that were not implanted with i.c.v. cannulas were sacrificed without receiving experimental treatment. Lysates were immunoblotted with anti-phospho ERK1/2 or anti-ERK1/2, and anti-phospho-CREB or anti-CREB antibodies. Following densitometry, intensities of bands corresponding to phosphorylated proteins were divided by intensities of the corresponding total protein bands to correct for small differences in protein loading. Results (mean ± S.E.M.) for caudate-putamen (top) and nucleus accumbens (bottom) are expressed in comparison to the normalized control, which was defined as the ad libitum fed group.

In rats without lateral ventricular cannulas that received no injection treatment prior to sacrifice, ERK1/2 phosphorylation did not differ between feeding groups (Figure 4; Cpu: t(8) = 1.2; NAc: t(8) = 1.0), nor did CREB phosphorylation (Cpu: t(8) = 1.2; NAc t(8) = 0.1).

**Figure 5:**
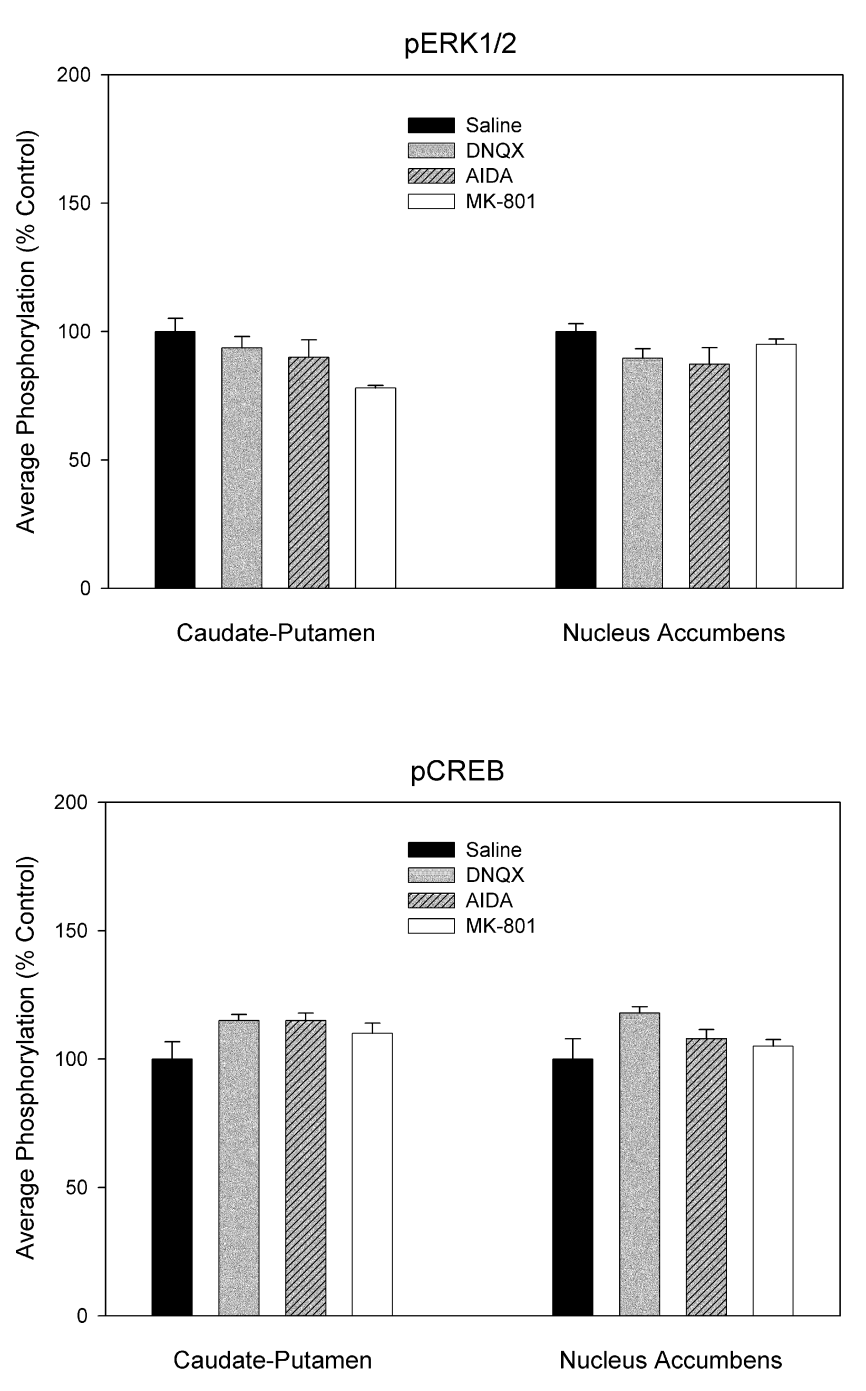
**Effects of Glutamate Receptor Antagonists on Activation of ERK1/2 and CREB in Caudate-Putamen and Nucleus Accumbens of Food-Restricted Rats **Food-restricted rats received i.c.v. injections of either saline vehicle, the AMPA antagonist DNQX (10 nmol), the group I mGluR antagonist AIDA (100 nmol), or NMDA antagonist MK-801 (100 nmol) prior to sacrifice. Lysates were immunoblotted with anti-phospho ERK1/2 or anti-ERK1/2 (top) and anti-phospho-CREB or anti-CREB (bottom) Following densitometry, intensities of bands corresponding to phosphorylated proteins were divided by intensities of the corresponding total protein bands to correct for small differences in protein loading. Results (mean ± S.E.M.) for caudate-putamen (left in each figure) and nucleus accumbens (right in each figure) are expressed in comparison to the normalized control, which was defined as the group injected with saline vehicle.

In food-restricted rats injected with GluR antagonists prior to sacrifice, neither ERK1/2 nor CREB phosphorylation differed from that observed in food-restricted rats injected with saline-vehicle (Figure 5).

## Discussion

Adding to the results of Choe and Wang who demonstrated DHPG-induced activation of ERK1/2 and CREB in dorsal striatum [12], the present study has demonstrated DHPG-induced activation of ERK/12 and CREB in both CPu and NAc following lateral ventricular infusion. While i.c.v. injection allows for the possibility that these effects were not directly mediated by striatal group I mGluRs, the proximity of striatum to the lateral ventrical and the high density of mGluRs in these structures support the likelihood of direct effects. Direct effects are also supported by the observed activation of Akt which mediates group I mGluR signal transduction [17].

As previously seen in rats challenged with the D-1 dopamine receptor agonist, SKF-82958, DHPG-induced activation of ERK1/2, CREB and c-Fos were greater in food-restricted than ad libitum fed rats. However, the pattern of results in this study – i.e. augmented ERK1/2 and CREB activation in food-restricted rats injected with vehicle, and no difference in Akt activation between feeding groups – suggests that group I mGluR signaling was not enhanced. Rather, a cellular-activating effect of the injection procedure was enhanced by food restriction and summated with the effect of DHPG. This response of vehicle-injected subjects was clearly a response to some aspect of the i.c.v. injection procedure because similarly food-restricted rats that were not challenged in any way prior to sacrifice displayed levels of pERK1/2 and pCREB that were essentially identical to those of ad libitum fed rats. Furthermore, the augmented activation of ERK1/2 and CREB in vehicle-injected food-restricted rats was not mediated by Akt because Akt, though activated by DHPG, did not differ between food-restricted and ad libitum fed rats injected with vehicle or DHPG.

Attribution of the augmented DHPG-induced ERK1/2 and CREB phosphorylation to a "nonspecific" response to i.c.v. injection distinguishes the group I mGluR from the D-1 DA receptor. Although a modestly enhanced ERK response to i.c.v. vehicle infusion was seen in the NAc of food-restricted rats in the prior study, the dramatically increased activation of ERK1/2 and CREB by SKF-82958 in CPu and NAc was dissociable from any response to the injection procedure [4]. Even mild stressors are known to stimulate DA [18] and Glu [19,20] release in striatum, and it is possible that food-restriction augments this physiological response or the cell signaling induced by it. Because the ionotropic (AMPA and NMDA) as well as group I mGluRs are abundant in striatum and all three receptor types mediate MAP kinase signaling [12,21,22], corresponding antagonists were injected i.c.v. and compared to vehicle. It was reasoned that if the physiological response to the injection procedure involves one of these GluRs, ERK1/2 and CREB phosphorylation would be decreased, relative to vehicle, in the group(s) receiving the corresponding antagonist. The MK-801 treatment was also a potential probe for mediation by D-1 DA receptors because D-1 DA agonist-induced activation of ERK1/2 and CREB are dependent on the NMDA receptor in food-restricted subjects [9]. Results of this test did not provide support for mediation by one of the GluR types. Because only one dose of each antagonist was used, these results must be considered preliminary. However, considering the proximity of striatum to the lateral ventricle, and the fact that the doses used have exerted measurable cellular, physiological or behavioral effects in other studies [23-26] there is some doubt about involvement of GluRs in the effect of "injection stress".

An interesting possibility to consider is mediation of the response by brain-derived neurotrophic factor (BDNF). Food-restricted rats have elevated striatal BDNF levels which appear to be involved in an enhanced neuroprotective response to diverse insults [27]. BDNF activates striatal ERK1/2 which, unlike glutamate-induced activation of Akt, ERK1/2 and CREB is not blocked by a PI 3-kinase inhibitor [21]. It is therefore possible that an enhanced BDNF-mediated activation of ERK1/2 and CREB in food-restricted rats represents a neuroprotective response to intraventricular infusion.

## Conclusions

The present results indicate that a group I mGluR agonist activates Akt, ERK1/2 and CREB in both the CPu and NAc. Further, activation of ERK1/2, CREB, and c-Fos are stronger in food-restricted than in ad libitum fed rats. The augmented response is not attributed to increased Group I mGluR function but, instead, to an augmented response of food-restricted rats to the i.c.v. injection procedure. Some evidence casting doubt on attribution of the latter response to GluRs was obtained. It will be of interest to evaluate whether striatal MAPK signaling in food-restricted rats is generally augmented in response to stressors or whether this response is peculiar to i.c.v. infusion. In addition, it will be of interest to evaluate whether this physiological response is mediated by BDNF and its TrkB receptor.

## Methods

### Subjects and surgery

All experimental procedures were approved by the New York University School of Medicine Institutional Animal Care and Use Committee and were performed in accordance with the "Principles of Laboratory Animal Care" (NIH publication number 85-23, revised 1996).

Subjects were male Sprague-Dawley rats (375–425 g) housed individually in plastic cages with free access to food and water except when food restriction conditions applied. Animals were maintained on a 12-h light/dark cycle, with lights on at 07:00 h. Rats were anesthetized with ketamine (100 mg/kg; i.p.) and xylazine (10 mg/kg; i.p.) and stereotaxically implanted with a 26-gauge guide cannula (Plastics One, Roanoke, VA USA) in the right lateral ventricle. The cannula was permanently affixed to the skull by flowing dental acrylic around it and four surrounding mounting screws. Patency of the guide cannula was maintained with an occlusion stylet. Several days after surgery, cannula placements were confirmed by demonstration of a vigorous and short latency (i.e. <60 s) drinking response to 50 ng of angiotensin II.

### Food restriction and habituation

Seven days following surgery, half the subjects were put on a food restriction regimen whereby a single 10 g meal of Purina (Gray Summit, MO USA) rat chow was delivered at approximately 17:00 h each day. Rats continued to have *ad libitum *access to water. Once body weight had declined by 20–25% (approximately 15 days) daily food allotments were titrated for an additional week to maintain stable body weight. During the 3 weeks required for food-restricted rats to attain and stabilize at target body weights, all rats were habituated, on five occasions, to the handling and injection procedures to be employed on the terminal day of the experiment.

### Drug treatment

In Experiment 1, six food-restricted and six ad libitum fed rats received i.c.v injections of sterile 0.9% saline (5 μl). Six food-restricted and six ad libitum fed rats received i.c.v injections of the group I metabotropic glutamate receptor agonist, DHPG (500 nmol in 5 μl; Tocris Cookson, Ellisville, MO, USA). An additional five food-restricted and four ad libitum fed rats received i.c.v. injections of DHPG and brains were processed for Fos-immunostaining. In Experiment 2, six unoperated food-restricted rats and six unoperated ad libitum fed rats were prepared and habituated as above and received no unusual handling or drug injection on the terminal day of the experiment. In the second part of Experiment 2, twenty four food-restricted rats with lateral ventricular cannulas were prepared and habituated as in Experiment 1. On the terminal day of the experiment, six were injected i.c.v. with saline vehicle (5.0 μl), six were injected with the AMPA glutamate receptor antagonist, DNQX (10 nmol; Sigma-Aldrich, St. Louis, MO), six were injected with the NMDA glutamate receptor antagonist, MK-801 (100 nmol; Sigma-Aldrich), and six were injected with the group I mGluR antagonist, AIDA (100 nmol; Tocris Cookson). Antagonist doses were chosen on the basis of prior findings [e.g. [23,26,28]] and/or being just below threshold for producing noticeable motoric abnormalities. For i.c.v injection, solutions were loaded into a 30 cm length of PE-50 tubing attached at one end to a 250-μl Hamilton syringe filled with distilled water and at the other end to a 33-gauge injector cannula which extended 1.0 mm beyond the implanted guide. The 5.0 μl injection volume was delivered over a period of 95 s. One minute following injection, internal cannulas were removed, stylets replaced, and animals were returned to home cages.

### Lysate preparation

Prior studies have indicated that MAP kinase activation is transient and the optimal time-point to study phosphorylation of ERK 1/2 after physiological or pharmacological treatment is 15–20 min [29]. Therefore, all rats, except the twelve unoperated/uninjected rats, were killed 20 min after injection by brief exposure to CO_2 _followed by decapitation. The twelve unoperated/uninjected rats were simply removed from home cages and exposed to CO_2 _followed by decapitation. Brains were rapidly removed and immediately frozen in powdered dry ice. Five hundred-micrometer sections were cut using an IEC Minotome cryostat, and CPu and NAc were micropunched, under an Olympus dissecting microscope, from a series of 8 consecutive frozen sections. The tissue was then homogenized in 10 volumes of 50 mM Tris-HCl, pH 7.5 containing 50 mM NaCl, 5 mM EDTA, 1 mM EGTA, 1 mM Na_3_VO_4_, 40 mM β-glycerophosphate, 50 mM NaF and 5 mM Na_4_P_2_O_7_, 1% Tx-100, 0.5 μM okadaic acid, 0.5% sodium deoxycholate and 0.1% SDS, followed by centrifugation and protein determination using BCA reagent kit as described by the manufacturer (Pierce) Supernatants were mixed with 5 × SDS-PAGE sample buffer, boiled for 5 min, cooled on ice and kept at -80°C until future use.

### Western blotting

Protein (10–30 μg per lane) was separated by electrophoresis on precast 10% polyacrylamide gels (Cambrex, East Rutherford, NJ, USA). Precision Plus protein standard molecular weight markers (Bio-Rad, Hercules, CA, USA) were also loaded to assure complete electrophoretic transfer and to estimate the size of bands of interest. The gels were transferred to nitrocellulose membrane (Osmonics) for 2 h, with a constant voltage of 100 volts. Membranes were blocked for 1 hr at room temperature with blocking buffer, 5% non fat dry milk in 50 mM Tris-HCl, pH 7.5 containing 150 mM NaCl and 0.1% Tween 20 (TBS-T), then probed overnight at 4°C using primary monoclonal antibodies for phospho-(Thr202/Tyr204)-p44/42 ERK1/2 (mouse monoclonal, 1:2000; Cell Signaling, Beverly, MA, USA), or polyclonal antibodies for phospho-Akt (Ser 473) (rabbit polyclonal, 1:1000 dilutuion, Cell Signaling), and phospho (Ser 133) CREB (rabbit polyclonal, 1:2000; Upstate Biotechnology, Lake Placid, NY, USA). Total levels of ERK1/2, Akt and CREB were detected on the same blots using anti-rabbit p42/44 ERK1/2 antibody 1:2000, (Cell Signaling), anti-rabbit total Akt (1:2000 dilution, Cell Signaling), or anti-rabbit CREB antibody (1:2000 dilution, Calbiochem). After detection of phosphorylated ERK1/2, phospho-Akt and CREB blots were stripped with 25 mM Glycine, pH 2.0 containing 1% SDS for 30 min at room temperature, washed six times in TBS-T buffer, blocked in blocking buffer for 1 h and then incubated overnight at 4°C in total ERK1/2, total Akt or CREB antibody. After probing with primary antibodies and washing with TBS-T buffer (3 × 5 min), membranes were incubated with 1:2000 dilution horseradish peroxidase conjugated anti-mouse or 1: 2000 dilution anti-rabbit IgG (Cell Signaling). Proteins were visualized using a chemiluminescense ECL kit (Pierce). Densitometric analysis of the bands was performed using NIH image software. Phospho-p42/44 MAPK, phospho Akt and phospho CREB values were normalized to total p42/44 MAPK, Akt and CREB values respectively.

### Immunohistochemistry

Ninety min after DHPG injection rats were anesthetized with sodium pentobarbital (50.0 mg/kg,i.p.) and transcardially perfused with isotonic phosphate buffered saline (PBS) followed by 4% paraformaldehyde in PBS. Brains were then removed and maintained in 20% sucrose at 4°C for 48 h. Forty μm sections were cut on an IEC Minotome cryostat and collected in a cryoprotective solution. Fos immunostaining was carried out using a rabbit polyclonal c-Fos antiserum (Oncogene Science-Calbiochem, La Jolla, CA) and the avidin-biotin peroxidase complex (ABC; Vector laboratories).

Sections were washed in 1% sodium borohydride followed by PBS and incubated for 2 hrs in 4% normal goat serum plus 1% BSA in PBS containing 0.2% Triton X-100 (Sigma-Aldrich) to block nonspecific binding. This was followed by incubation, overnight, with rabbit polyclonal c-fos antiserum (1:5000 dilution). Following several PBS washes, sections were incubated with a secondary antiserum (Vector, Burlingame, CA) for 60 min and subsequently reacted with avidin-biotin complex (ABC) (Vector). The peroxidase reaction was visualized with a chromogen solution containing 100 mM nickel sulfate, 125 mM sodium acetate, 10 mM imidazole, 0.03% diaminobenzidine (DAB), and 0.01% hydrogen peroxide at pH 6.5. Sections were then mounted on chrome-alum coated slides, dehydrated, and coverslipped.

Objective counting of c-Fos positive cells in CPu at coronal levels +1.7 and -0.3 mm, and NAc core and shell at coronal level +1.5 mm in relation to bregma [30] was accomplished with a light microscope (Olympus, CK2) equipped with a Sony XC-77 video camera module coupled to an MCID image analysis system (Imaging Research Inc., St. Catherines, Canada). For each region, bilateral grain counts from three to five consecutive sections were measured to arrive at an average bilateral value per rat. Because results obtained from anterior and posterior levels of CPu were essentially the same, results from the two levels were combined and averaged for each rat.

### Data analysis

For each Western blot, film exposure time was set as needed to visualize distinct bands in the control samples of each experiment. Immunoblots were analyzed using NIH imaging software. For each blot, relative phospho-protein levels were calculated from the ratio of optical density of the phosphorylated protein/total protein to correct for small differences in protein loading. In addition, tubulin levels were analyzed in several representative gels and no differences were observed between treatment groups. Results were expressed by comparison to the normalized control, which in Experiment 1 was defined as the ad libitum fed group injected with vehicle. In the first part of Experiment 2, unoperated/uninjected ad libitum fed rats served as control. In the second part of Experiment 2, food-restricted rats injected with i.c.v. saline vehicle served as control. Differences between treatment conditions in Experiment 1 were analyzed by two-way analysis of variance (ANOVA; feeding condition × injection treatment). Differences between treatment conditions in the first and second parts of Experiment 2 were analyzed by student's t-test and one-way ANOVA, respectively.

## Authors' contributions

YP conducted the majority of immunoblotting experiments plus the immunohistochemistry experiment, contributed to experimental design and assisted in manuscript preparation. YB provided technical supervision of immunoblotting experiments and assisted in manuscript preparation. KC contributed to design of the study, assisted in all experiments, and wrote the final draft of the manuscript.
